# Differences in Dialysis Efficacy Have Limited Effects on Protein-Bound Uremic Toxins Plasma Levels over Time

**DOI:** 10.3390/toxins11010047

**Published:** 2019-01-16

**Authors:** Detlef H. Krieter, Simon Kerwagen, Marieke Rüth, Horst-Dieter Lemke, Christoph Wanner

**Affiliations:** 1 Division of Nephrology, University Hospital Würzburg, 97080 Würzburg, Germany; simonkerwagen@gmx.de (S.K.); wanner_c@ukw.de (C.W.); 2 eXcorLab GmbH, 63785 Obernburg, Germany; marieke.rueth@excorlab.de (M.R.); horstdieter.lemke@excorlab.de (H.-D.L.)

**Keywords:** protein-bound uremic toxins, end-stage renal disease, hemodialysis, hemodiafiltration, dialysis adequacy

## Abstract

The protein-bound uremic toxins para-cresyl sulfate (pCS) and indoxyl sulfate (IS) are associated with cardiovascular disease in chronic renal failure, but the effect of different dialysis procedures on their plasma levels over time is poorly studied. The present prospective, randomized, cross-over trial tested dialysis efficacy and monitored pre-treatment pCS and IS concentrations in 15 patients on low-flux and high-flux hemodialysis and high-convective volume postdilution hemodiafiltration over six weeks each. Although hemodiafiltration achieved by far the highest toxin removal, only the mean total IS level was decreased at week three (16.6 ± 12.1 mg/L) compared to baseline (18.9 ± 13.0 mg/L, *p* = 0.027) and to low-flux dialysis (20.0 ± 12.7 mg/L, *p* = 0.021). At week six, the total IS concentration in hemodiafiltration reached the initial values again. Concentrations of free IS and free and total pCS remained unaltered. Highest beta_2_-microglobulin elimination in hemodiafiltration (*p* < 0.001) led to a persistent decrease of the plasma levels at week three and six (each *p* < 0.001). In contrast, absent removal in low-flux dialysis resulted in rising beta_2_-microglobulin concentrations (*p* < 0.001). In conclusion, this trial demonstrated that even large differences in instantaneous protein-bound toxin removal by current extracorporeal dialysis techniques may have only limited impact on IS and pCS plasma levels in the longer term.

## 1. Introduction

In chronic renal failure, the progressing accumulation of uremic toxins represents a hallmark of the uremic syndrome and causes associated morbidity and mortality [1]. Of the different classes of uremic toxins defined, protein-bound uremic toxins (PBT) are particularly difficult to remove by current dialysis procedures. Although most of the PBT are small substances, their binding to larger protein, particularly albumin, restrains them from freely passing through dialysis membranes [2]. Thus, only their much smaller free fraction, which is in equilibrium with the bound toxin, is able to be readily removed.

Several recent approaches aimed to increase PBT removal during extracorporeal dialysis therapy, either by interfering with the protein-binding [3,4] or by adding adsorptive components [5,6,7]. But also simply modifying routine dialysis treatment is suited to significantly improve PBT elimination. This can be achieved for example by longer sessions, by increasing the dialysis membrane surface area and the blood and dialysate flow rate as well as by enhancing convective forces, i.e., switching from hemodialysis to highly efficient hemodiafiltration [8,9,10,11].

Para-cresyl sulfate (pCS) and indoxyl sulfate (IS) represent prototypical substances of uremic toxins highly bound to albumin (>90%). Both are associated with all-cause mortality in chronic kidney disease [12]. While instantaneous and short-term effects of the different routine and experimental dialysis techniques on pCS and IS plasma levels have been repeatedly investigated, only very few trials have focused on their longer-term variations [10,13,14]. These studies, which largely differed in design and execution, showed inconsistent results with regard to the PBT plasma concentrations.

The purpose of the present clinical trial was to demonstrate the effects of three extracorporeal dialysis forms, differing in treatment efficacy, on pCS and IS plasma levels over a six-week period.

## 2. Results

Of the 15 patients enrolled, one patient had to be excluded due to being hospitalized. Fourteen patients (67.7 ± 12.0 years; 10 male, 4 female) terminated the study and were analyzed as per protocol. All patients had patent arterio-venous fistulae and were on a high-flux dialyzer for at least one year prior to the trial. Their underlying renal diseases were glomerulonephritis (*n* = 4), diabetic nephropathy (*n* = 4), hypertensive nephropathy (*n* = 2), urate nephropathy (*n* = 1), tubulo-interstitial nephritis (*n* = 1), renal ischemia due to ruptured aortic aneurysm (*n* = 1), and unknown (*n* = 1). The dialysis vintage was 84.6 ± 28.0 months and only 5 of the 14 patients had residual renal function. At baseline, none of the patients presented symptoms of ongoing infection, although, microinflammation was present as indicated by slightly elevated CRP values (refer to Table 1).

Treatment time in each group was identical and lasted 268 ± 17 min. Blood flow rates were not different, being 381 ± 30, 379 ± 33, and 385 ± 25 mL/min for low-flux hemodialysis (LFHD), high-flux hemodialysis (HFHD), and hemodiafiltration (HDF), respectively. Ultrafiltration volumes were similar with 3002 ± 1119, 3033 ± 1180, and 2845 ± 1180 mL, respectively. In HDF, the convective volume averaged at 24.3 ± 4.9 L.

### 2.1. Treatment Effects on Study End-Points

#### 2.1.1. Plasma Concentrations of Toxins over Time

**Figure 1:**
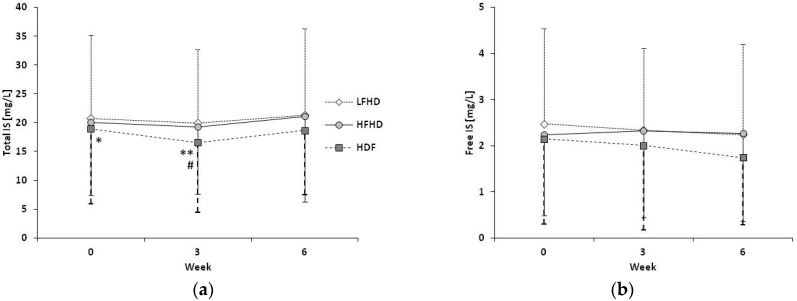
Course of mean indoxyl sulfate (IS) plasma levels ± standard deviations over the six-week study period: (**a**) Total IS. During hemodiafiltration (HDF), concentrations decreased until week 3. At t0 and t3, they were lower compared to low-flux hemodialysis (LFHD). * *p* = 0.046, ** *p* = 0.021 vs. low-flux hemodialysis (HD). ^#^ *p* = 0.027 vs. t0; (**b**) Free IS. No significant differences between and within dialysis treatments were observed.

**Figure 2:**
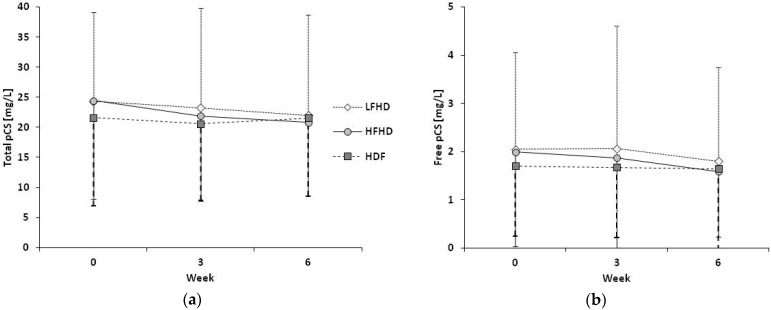
Course of para-cresyl sulfate (pCS) plasma levels over the six-week study period. Data are mean values ± standard deviations. No differences between and within dialysis treatments were detected: (**a**) Total pCS; (**b**) Free pCS.

During HDF, plasma concentrations of total IS decreased from baseline (t0, 18.9 ± 13.0 mg/L) to week 3 (t3, 16.6 ± 12.1 mg/L, *p* = 0.027) (refer to Figure 1a). Total IS levels at t0 and t3 in patients on HDF were lower compared to LFHD (20.8 ± 14.4 mg/L, *p* = 0.046, and 20.0 ± 12.7 mg/L, *p* =0.021, resp.), but did not differ from HFHD (20.1 ± 12.6 and 19.3 ± 11.6 mg/L). In contrast, plasma levels of free IS and pCS as well as of total pCS were not different, neither within nor between treatment modes (refer to Figure 1b and Figure 2).

The differences in beta_2_-microglobulin (b2M) removal had significant impact on b2M plasma concentrations. Compared to baseline, the levels in LFHD were increased at week 3 and week 6 (*p* < 0.001). At these times, they were also much higher compared to both HFHD and HDF (*p* < 0.001). Only in HDF, the b2M levels at week 3 and week 6 were reduced (*p* < 0.05) compared to baseline. No change was noted in HFHD (refer to Table 2).

#### 2.1.2. Instantaneous Treatment Efficacy

Total IS reduction ratios in HDF (t0, 48.1 ± 9.6; t3, 46.0 ± 18.3%) were much higher (*p* < 0.001) compared to low-flux (36.5 ± 10.7; 34.3 ± 8.2%) and high-flux HD (35.9 ± 8.7; 37.0 ± 10.5%), as shown in Table 3. The same trend was observed for the reduction ratios of total pCS. Free IS and free pCS reduction ratios at week 0 were also significantly higher in HDF compared to LFHD and HFHD (*p* < 0.001). At week 3, this difference could only be determined versus LFHD (refer to Table 3).

In HDF, higher dialytic clearances of total pCS and total IS were only noted at week 0 compared to LFHD, (each *p* < 0.05) (refer to Table 3). The dialytic clearance of total IS in HFHD was also higher compared to LFHD at week 0 (*p* < 0.05). As Table 3 shows, the masses of the protein-bound toxins pCS and IS in dialysate were not different between treatments. 

The reduction ratios of the middle molecular solute b2M in HDF at week 0 and week 3 were significantly higher (each *p* < 0.001) compared to HFHD and LFHD. Reduction ratios in HFHD were also superior compared to LFHD (each *p* < 0.001). The same differences between the treatment modes were also determined for the dialytic clearances and removed masses in dialysate of b2M (refer to Table 3).

Differences in treatment efficacy were also seen with regard to the single pool Kt/V for urea (spKt/V) as a measure of small solute dialysis dose. Compared to both LFHD and HFHD, HDF achieved considerably higher spKt/V values at week 0 and week 3 (each *p* < 0.001). Furthermore, at week 0, the dialysis dose in HFHD was superior to LFHD (*p* < 0.001) (refer to Table 3).

#### 2.1.3. Other Laboratory Parameters

No significant differences in CRP levels were detected either within or between treatments (refer to Table 2), indicating that the dialysis mode had no impact on this parameter.

At week 3, the pre-treatment plasma albumin concentration in patients on HDF was lower compared to baseline (*p* < 0.05) as well as to LFHD and HFHD (*p* < 0.01) (refer to Table 2). The level recovered until week 6 in the further course of the study. In contrast, a significant increase of the albumin level from baseline until week 6 was noted for LFHD (*p* < 0.05).

#### 2.1.4. Associations between Parameters

**Figure 3:**
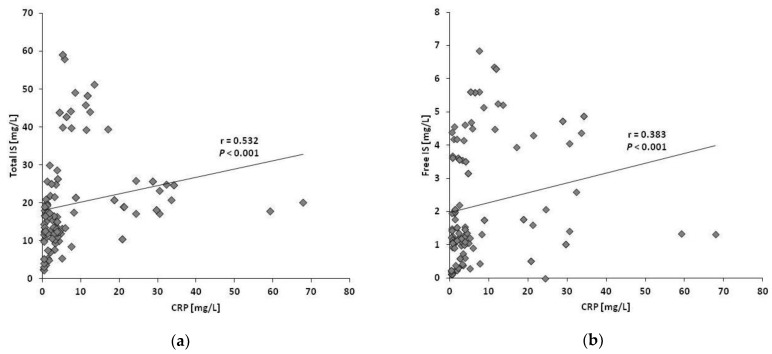
Association of IS plasma concentrations with CRP. Both total and free IS highly correlated with CRP: (**a**) Total IS vs. CRP; (**b**) Free IS vs. CRP.

Levels of free and total PBT correlated with each other. Interestingly, free and total IS concentrations in plasma were positively associated with CRP (*r* = 0.383 and 0.532, resp.; each *p* < 0.001) (refer to Figure 3), but not with albumin (*r* = 0.075 and 0.129, resp.; *p* = 0.401 and 0.149). In contrast, within the different treatment forms, PBT levels did not correlate with CRP. Furthermore, associations between albumin and PBT were also absent when analyzed within the different dialysis modes (LF-HD, HF-HD and HDF; free IS, *r* = −0.149, 0.018 and −0.115, resp.; *p* = 0.346, 0.908 and 0.467; total IS, *r* = 0.104, 0.172 and 0.007, resp.; *p* = 0.511, 0.275 and 0.946; free pCS, *r* = 0.016, 0.022 and −0.020, resp.; *p* = 0.922, 0.888 and 0.901; total pCS, *r* = 0.068, 0.091 and 0.129, resp.; *p* = 0.669, 0.566 and 0.417). 

Small and particularly middle molecule removal in dialysis was significantly associated with PBT elimination. The reduction ratios of total pCS (*r* = 0.620) and total IS (*r* =0.649) correlated significantly (*p* < 0.001) with spKt/V. The reduction ratio of b2M correlated with those of the free and total fractions of PBT (free pCS *r* = 0.389, total pCS *r* = 0.501, free IS *r* = 0.369, total IS *r* = 0.530; each *p* < 0.001).

## 3. Discussion

The present study demonstrated that large differences in the elimination of the highly protein-bound toxins pCS and IS during dialysis had only very limited impact on their pre-treatment plasma levels over a six-week period. High convective volume HDF, performed according to the current recommendations [15], achieved by far higher PBT removal than LFHD and HFHD, both deliberately executed in a standard manner. The superior elimination of PBT in HDF resulted only in a slight and transient decrease (−12%) of the total IS level after three weeks of treatment. At this time, the level was also lower compared to LFHD. However, at week 6, differences between baseline values and therapy modes had disappeared. For pre-treatment pCS concentrations, no differences were determined at all.

These results only partially match those of previous similar trials. They basically confirm a recent crossover study by Camacho et al., who also aimed to apply dialysis therapy varying in efficacy for a limited period of time [10]. In 14 patients on thrice-weekly six to eight hour nocturnal therapy, hemodialysis was modified to provide one 2-week period of high and another 2-week period of low PBT clearance separated by a wash-out period of several weeks. Pre-treatment free as well as total IS levels were slightly decreased (−11 and −4%, respectively) at the end of the high clearance period, while they were not in the low clearance group. Despite large differences in pCS removal between therapy groups, no change in pre-treatment free and total pCS plasma levels was achieved. The different courses of pre-treatment IS and pCS were explained by the short experimental periods of only two weeks and an increase in intestinal pCS generation in response to higher clearance [10]. 

An earlier trial was able to show that, compared to HFHD and low-efficient predilution HDF, two weeks of intensified convective treatment in form of predilution HDF with 60L infusion volume could reduce pre-treatment para-cresol plasma levels [16]. The same study did not find a change of para-cresol plasma levels when postdilution HDF, the convective therapy mode of the present testing, was performed. With 20L of infusion fluid, the total convective volume was slightly lower compared to that of the present study. Although a randomized, prospective, crossover design was used [16], the results must be regarded with some caution because para-cresol and not only pCS was measured. Para-cresol includes pCS and para-cresyl glucuronide, the latter is only little bound to protein and, therefore, easily removed by dialysis in contrast to pCS [9].

Meert et al. performed a study with a longer observation period, which, unfortunately, was compromised by a non-randomized study design. They demonstrated a consistent 20% reduction of pCS over nine weeks after 13 maintenance dialysis patients were switched from HFHD to online postdilution HDF with a total convective volume of 21.7 L [13]. The results were in line with findings from a previous longitudinal study, which did a post hoc analysis on remnant samples after six months of predilution hemofiltration [17].

The trial with the longest observation period was the recent Italian REDERT study, which compared six months of randomly assigned LFHD to six months of postdilution HDF in a crossover manner. A total of 36 patients were analyzed and the mean convective volume in HDF was 23.8 ± 2.3 L, i.e., only slightly lower as in the present trial. At the end of the respective six-month periods, pretreatment levels of free and total pCS and IS were significantly lower in HDF compared to LFHD [14].

Comparing the data generated in the present trial with those of the references raises several questions. In the present study, the elimination of PBT highly correlated with treatment efficacy, particularly for small solutes measured as spKt/V for urea, but also for middle molecules with the well-known lowering impact on the course of the pretreatment b2m levels in plasma over time (refer to Table 3) [13,18,19]. A correlation of IS and pCS removal with spKt/V has been reported before [11]. In the REDERT study, the difference in spKt/V between LFHD and HDF was not large (1.36 vs. 1.49) [14], suggesting that this could have been also true for PBT elimination, which, however, was not measured. Furthermore, dialysis dose assessed as Kt/V seems to be no determinant for pre-treatment PBT concentrations, but other factors, such as residual renal function and dietary protein intake, are highly associated with the levels [20]. In fact, vegetarian patients on maintenance hemodiafiltration have lower PBT levels than those on a mixed diet [21], while casual changes in nutrition most likely cause the spontaneous variations of pre-dialysis uremic toxin concentrations, which are observed in otherwise stable patients on maintenance hemodialysis [22]. Therefore, it appears obvious that all studies investigating plasma PBT levels over time, including the present one, may have been confounded by the patients’ residual renal function and, particularly, diet, as they were not controlled for either.

Switching a patient from HD to HDF frequently leads to a slight, usually transient reduction in serum albumin concentrations [19,23], an observation also made in the present trial despite a very limited mean albumin loss of 1.4 ± 0.5 g into dialysate per session. In LFHD and HFHD, the albumin loss was below the detection limit of 0.3 g in almost all treatments and serum albumin even increased in LFHD. The transient serum albumin reduction in HDF at week 3 paralleled the decrease of IS. Although IS, as well as pCS, is mainly bound to albumin [24], lowering albumin does not fully explain the reduction of IS because the concentrations of the two substances did not correlate. It is likely that a reduced albumin level in HDF is compensated by an increase of albumin synthesis, which requires more protein intake [25]. In fact, such a dietary adjustment may have confounded the beneficial effects on the PBT plasma levels by the superior elimination in this dialysis mode. In this context, it needs to be mentioned that inflammation is known to adversely affect albumin synthesis [25], and, in this small present trial, a positive association of CRP and IS plasma levels has been determined. Although both IS and inflammation are involved in cardiovascular outcomes in patients with chronic renal failure stage 3-5 [12], such an association has not been described previously [26].

IS and pCS derive from the metabolism of aromatic amino acids, more specifically the essential dietary tryptophan and tyrosine and phenylalanine, respectively, after bacterial degradation in the intestine, from where they are adsorbed [27]. Therefore, modifying the gut microbiom and interfering with toxin and toxin precursor adsorption represent conclusive therapeutic interventions to reduce uremic toxin accumulation in chronic renal failure. In this regard, modifications of diet, applications of prebiotics and/or probiotics, as well as ingestion of oral intestinal sorbents have proven therapeutic effectiveness [27], emphasizing casual changes in nutrition and PBT resorption from the intestine as possible confounders in the present trial. 

IS and pCS are normally cleared by renal tubular secretion with clearances higher than the glomerular filtration rate [28]. Therefore, it will also be interesting to study the effects of PBT removal by future bioartificial kidneys, which add renal tubular functions to dialysis therapy [29]. However, six weeks are a rather short observation period and in case of longer-term monitoring, a reduction of the PBT levels, such as in the previous studies [13,14], could have been occurred also in the present trial.

## 4. Conclusions

Extracorporeal dialysis therapies performed according to current guidelines highly differ in PBT elimination. Despite by far superior removal of pCS and IS in HDF, a sustained effect on PBT plasma levels after six weeks was not observed. Therefore, differences of current dialysis techniques in instantaneous PBT removal have only limited impact on their plasma levels in the longer term. Although more efficient dialysis strategies to increase PBT elimination may be available in the future [4,5,6,7], other determinants of PBT plasma concentrations, such as the intestinal PBT generation and adsorption, represent important therapeutic targets. In this regard, the oral administration of the charcoal adsorbent AST-120 has proven as an efficient example of lowering PBT levels in patients on maintenance hemodialysis [30].

## 5. Materials and Methods

The study was performed in adherence to the Declaration of Helsinki. Study approval was given by the Ethics Committee of the University hospital Würzburg (registration no. 66/16, date of the approval: 31 May 2016). The study was registered at the German Register for Clinical Trials (DRKS00010788). Written informed consent, which included the approval for publication of the study data, was obtained from all patients participating in the study. 

### 5.1. Study Design

**Figure 4:**
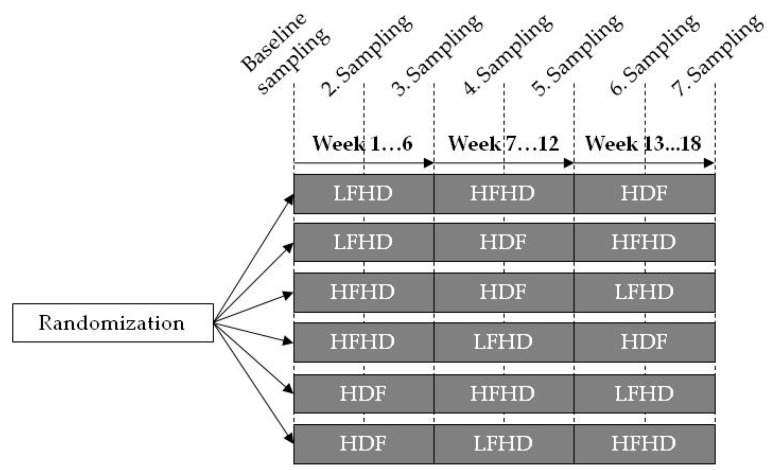
Study flow chart. Two or three patients each were randomly assigned to receiving one of six possible sequences of three different consecutive dialysis treatment forms each lasting for six weeks. A total of 15 patients were randomized. Blood and dialysate samples were drawn at the beginning, after three weeks and at the end of each treatment period. LFHD, low-flux hemodialysis; HFHD, high-flux hemodialysis; HDF, hemodiafiltration.

In a prospective, controlled, cross-over trial, 15 maintenance dialysis patients were enrolled. Each patient was randomly subjected to thrice weekly LFHD, HFHD, and high convective volume (study target ≥ 25 L) postdilution HDF, each for six consecutive weeks starting on a midweek session (refer to Figure 4). Dialysis membrane material was always identical (polyethersulfone/polyvinylpyrrolidone blend, PUREMA^®^, 3M^TM^ Deutschland GmbH, Wuppertal, Germany), but permeability and surface area were adapted to the dialysis mode (LFHD, PUREMA^®^ L 1.4 m²; HFHD, PUREMA^®^ H 1.4 m²; and HDF, PUREMA^®^ H 1.8 m²). Ultrapure dialysate was applied exclusively. The dialysate was composed as follows: Na^+^ 138 mmol/L; K^+^ 2–4 mmol/L, adapted according to the patients’ requirements; Mg^2+^ 0.5 mmol/L; Ca^2+^ 1.75 mmol/L; CH_3_COO^−^ 3.0 mmol/L; Cl^−^ 110.5 mmol/L; HCO_3_^−^ 32.0 mmol/L; glucose 1.0 g/L. Dialysate flow rates varied between HD and HDF (500 vs. 700 mL/min). Blood flow rates and treatment time were kept identical for individual patients, but differed between patients. Anticoagulation with standard (*n* = 12) or fractionated (*n* = 2) heparin was unchanged adopted from the patients’ routinely used regimen. 

### 5.2. Monitoring of Treatment Effects

Plasma levels of free and total pCS and IS were determined at baseline (t0), after three (t3) and six weeks (t6) of each treatment period. Reduction ratios, dialytic clearances, and mass removed into dialysate of PBT and b2M as well as spKt/V for urea were measured as reported previously at the t0 and t3 midweek treatments. Reduction ratios were calculated after correction of the venous blood value for extracellular volume changes [4]. Single-pool Kt/V was calculated using the second generation logarithmic estimate by Daugirdas [31]. Serum CRP and albumin were also monitored at t0, t3, and t6. Blood samples were drawn from the arterial needle before the treatment and, if applicable, from the venous blood line at the end of dialysis after reducing the blood flow rate to 50 mL/min and the dialysate flow turned off for 30 s. 

### 5.3. Analytical Methods

IS and pCS were measured by reversed-phase high performance liquid chromatography as previously described [32]. The purified IS (Sigma, Taufkirchen, Germany) and pCS (kindly provided by Natalie Meert, University of Ghent) standards equaled coeluting peaks derived from plasma of uremic individuals by mass spectroscopy (HCT IonTrap LC/MS-system; Bruker Daltronic GmbH, Bremen, Germany). Albumin, CRP and b2M were measured using laser nephelometry (BN ProSpec, Siemens, Germany). 

### 5.4. Data Analysis

Descriptive analysis of the results was performed by calculating mean values ± standard deviations (SD). Within-subject between-treatment differences and within-subject within-treatment changes from baseline were analyzed in series by ANOVA and a Tukey post hoc test for normally distributed samples. The Kruskal–Wallis and Spearman tests were used if normal distribution did not apply. A *p*-value of <0.05 was considered statistically significant. The statistical analysis was performed with the ‘Minitab 17 Statistical Software’ package (Minitab, Inc., State College, PA, USA, 2014).

## Figures and Tables

**Table 1:** Baseline characteristics of the patients analyzed as per protocol (*n* = 14).

Characteristic	Result
Age—years	67.7 ± 12.0
Female sex—no. (%)	4 (28.6)
Renal disease—no. (%)	
Glomerulonephritis	4 (28.6)
Diabetic nephropathy	4 (28.6)
Hypertensive nephropathy	2 (14.3)
Urate nephropathy	1 (7.1)
Tubulo-interstitial nephritis	1 (7.1)
Renal ischemia	1 (7.1)
Unknown	1 (7.1)
Dialysis vintage—months	84.6 ± 28.0
Smoker—no. (%)	3 (21.4)
Residual renal function—no. (%)	5 (35.7)
Diabetes mellitus—no. (%)	5 (35.7)
History of cardiovascular disease—no. (%)	
Myocardial infarction	2 (14.3)
Coronary heart disease	3 (21.4)
Congestive heart failure	1 (7.1)
Cardiac valve disorder	1 (7.1)
Peripheral vascular disease	8 (57.1)
Stroke or transient ischemic attack	2 (14.3)
Body weight—kg	79.5 ± 6.4
Hemoglobin—g/dl	11.4 ± 0.7
Albumin—g/L	34.7 ± 2.7
C-reactive protein—mg/L	6.4 ± 8.9
Calcium—mmol/L	2.34 ± 0.17
Phosphate—mmol/L	1.41 ± 0.41

**Table 2:** Time course of C-reactive protein (CRP), albumin, and beta_2_-microglobulin (b2M) plasma concentrations. Mean values ± standard deviations are given.

	CRP [mg/L]			Albumin [g/L]			b2M [mg/L]		
	Week 0	Week 3	Week 6	Week 0	Week 3	Week 6	Week 0	Week 3	Week 6
LFHD	7.1 ± 9.0	7.9 ± 9.9	11.4 ± 19.2	36.0 ± 4.0	35.8 ± 3.8 ^1^	38.2 ± 4.9 ^2^	23.7 ± 7.4	34.0 ± 13.5 ^4^	35.7 ± 14.4 ^4^
HFHD	7.1 ± 8.8	9.7 ± 16.5	7.7 ± 10.4	35.5 ± 3.3	34.9 ± 3.4 ^1^	37.1 ± 4.3	27.1 ± 11.9	23.8 ± 7.7	24.5 ± 7.4
HDF	7.1 ± 10.8	7.5 ± 9.2	5.1 ± 7.2	35.5 ± 4.7	33.8 ± 3.8 ^3^	36.7 ± 4.7	28.7 ± 13.1	22.2 ± 6.4 ^2^	22.4 ± 6.2 ^2^

^1^*p* = 0.01 vs. HDF; ^2^ *p* < 0.05 vs. Week 0; ^3^ *p* < 0.001 vs. Week 0; ^4^ *p* < 0.001 vs. Week 0 and vs. high-flux hemodialysis (HFHD) and hemodiafiltration (HDF).

**Table 3:** Instantaneous treatment efficacy (plasma reduction ratio, dialytic clearance, mass removed into dialysate, spKt/V) of the dialysis procedures at baseline and week 3. Mean values ± standard deviations are shown.

Parameter	LFHD		HFHD		HDF	
	Week 0	Week 3	Week 0	Week 3	Week 0	Week 3
Reduction Ratio [%]						
total IS	36.5 ± 10.7	34.3 ± 8.2	35.9 ± 8.7	37.0 ± 10.5	48.1 ± 9.6 ^1^	46.0 ± 18.3 ^1^
free IS	48.3 ± 11.6	44.0 ± 16.1	49.8 ± 12.7	49.4 ± 20.0	60.8 ± 10.4 ^1^	56.3 ± 23.1 ^2^
total pCS	30.7 ± 11.9	29.4 ± 7.3	29.7 ± 10.8	30.8 ± 10.6	42.8 ± 10.0 ^1^	39.1 ± 20.6 ^1^
free pCS	43.5 ± 13.9	40.0 ± 16.4	47.1 ± 13.6	47.8 ± 18.6	58.9 ± 10.6 ^1^	52.3 ± 25.9 ^2^
b2M	−1.9 ± 8.0	−0.3 ± 6.7	60.6 ± 6.0 ^3^	60.6 ± 6.0 ^3^	77.9 ± 4.9 ^1^	78.4 ± 5.1 ^1^
Dialytic Clearance [mL/min]						
total IS	20 ± 16	21 ± 14	25 ± 18 ^4^	26 ± 24	30 ± 23 ^4^	23 ± 23
total pCS	18 ± 16	18 ± 16	24 ± 19	23 ± 21	26 ± 18 ^4^	25 ± 19
b2M	8 ± 0	8 ± 1	33 ± 19 ^3^	33 ± 20 ^3^	52 ± 27 ^1^	49 ± 24 ^1^
Mass in Dialysate [mg]						
total IS	73 ± 64	76 ± 57	93 ± 72	97 ± 97	110 ± 91	89 ± 90
total pCS	70 ± 65	70 ± 66	92 ± 80	87 ± 84	96 ± 72	92 ± 76
b2M	30 ± 2	30 ± 2	124 ± 78 ^3^	121 ± 78 ^2^	190 ± 108 ^1^	174 ± 87 ^2^
spKt/V						
urea	1.53 ± 0.31	1.59 ± 0.29	1.69 ± 0.27 ^3^	1.68 ± 0.35	2.01 ± 0.35 ^1^	2.03 ± 0.33 ^1^

^1^*p* < 0.001 vs. LFHD and HFHD; ^2^ *p* < 0.01 vs. LFHD; ^3^ *p* < 0.001 vs. LFHD; ^4^ *p* < 0.05 vs. LFHD.
